# A Review of External Confinement Methods for Enhancing the Strength of Concrete Columns

**DOI:** 10.3390/ma18143222

**Published:** 2025-07-08

**Authors:** Oliwia Sikora, Krzysztof Adam Ostrowski

**Affiliations:** 1 Cracow University of Technology, CUT Doctoral School, Faculty of Civil Engineering, 24 Warszawska Str., 31-155 Cracow, Poland; 2 Cracow University of Technology, Faculty of Civil Engineering, 24 Warszawska Str., 31-155 Cracow, Poland

**Keywords:** concrete columns, FRP composite, strengthening, confinement, structural strengthening

## Abstract

The growing application of carbon fiber-reinforced polymers (CFRPs) in construction opens new possibilities for replacing traditional materials such as steel, particularly in strengthening and retrofitting concrete structures. CFRP materials offer notable advantages, including high tensile strength, low self-weight, corrosion resistance, and the ability to be tailored to complex geometries. This paper provides a comprehensive review of current technologies used to strengthen concrete columns, with a particular focus on the application of fiber-reinforced polymer (FRP) tubes in composite column systems. The manufacturing processes of FRP composites are discussed, emphasizing the influence of resin types and fabrication methods on the mechanical properties and durability of composite elements. This review also analyzes how factors such as fiber type, orientation, thickness, and application method affect the load-bearing capacity of both newly constructed and retrofitted damaged concrete elements. Furthermore, the paper identifies research gaps concerning the use of perforated CFRP tubes as internal reinforcement components. Considering the increasing interest in innovative column strengthening methods, this paper highlights future research directions, particularly the application of perforated CFRP tubes combined with external composite strengthening and self-compacting concrete (SCC).

## 1. Introduction

The historical development of concrete columns reflects major advances in engineering aimed at improving structural performance and durability. Since the introduction of reinforced concrete in the 19th century, which combined the compressive strength of concrete with the tensile capacity of steel, slender and efficient columns became feasible. This innovation played a key role in enabling taller structures and longer spans, influencing urban design. Early 20th-century applications demonstrated the reliability and longevity of reinforced concrete elements [1]. Research also highlighted how different loading histories affect the performance limits of circular bridge columns, especially their displacement behavior under repeated loads [2].

Prestressed concrete, introduced and widely adopted in the mid-20th century, further advanced column design by enabling reduced cross-sectional dimensions and increased strength and stiffness [3]. However, long-term use revealed challenges, particularly related to the corrosion of tendons and the loss of prestress due to moisture and chloride exposure [4,5]. In response, alternative prestressing methods have been explored such as the use of Fe-Mn-Al-Ni shape memory alloy (SMA) bars in Ultra-High Performance Concrete (UHPC), which have shown improved flexural performance after thermal activation [6]. Prestraining and thermal activation of the shape memory alloys significantly improved the maximum flexural strength, demonstrating the potential for such systems to address durability and performance issues in prestressed concrete structures.

**Figure 1:**
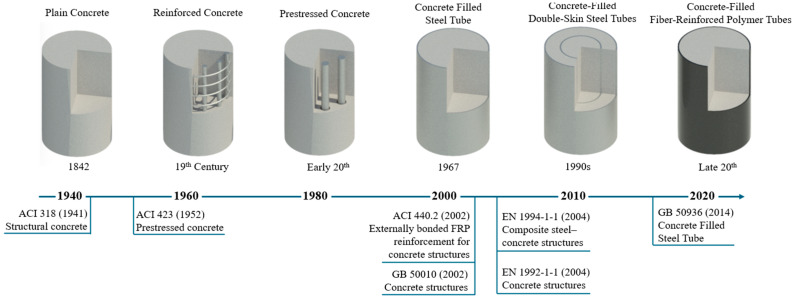
Historical development of concrete structural systems alongside the timeline of related design standards [20,21,22,23,24,25,26].

In recent decades, alongside traditional methods of strengthening reinforced concrete columns—such as increasing the cross-section through additional reinforcement or applying prestressed reinforcement—alternative techniques have also been developed that significantly expand the range of available engineering solutions. One such approach involves Concrete-Filled Steel Tube (CFST) columns, in which a concrete core is encased within a steel tube. The combination of these two materials enables favorable interaction: the steel jacket carries tensile stresses and provides effective confinement of the concrete, which enhances its compressive strength [7,8]. CFST structures are characterized by high load-bearing capacity, increased fire resistance, and favorable behavior under dynamic loads, making them an attractive solution in many engineering applications [9,10,11]. Another rapidly developing strengthening method involves polymer matrix composite materials, known as Fiber-Reinforced Polymers (FRPs) [12,13,14]. These materials, owing to their high strength/weight ratio, corrosion resistance, and ease of application, are widely used in the strengthening of concrete structures [15,16,17]. Typical application methods include wrapping structural elements with composite sheets or fabrics, which enhances axial load capacity, shear strength, and fatigue resistance of columns. An additional advantage of FRP is the ability to precisely tailor the strengthening characteristics to structural requirements by selecting the cross-section, type of fibers (carbon, glass, aramid), the number of layers, and the fiber orientation [18,19]. The key stages in the development of concrete structural systems, along with the corresponding design standards, are summarized in Figure 1. Owing to these features, the subject of strengthening concrete columns with composite materials constitutes the main focus of this study, in which the mechanisms of action, application methods, and effectiveness of such reinforcements will be discussed in detail.

To provide a comprehensive overview of the structural, economic, and environmental implications of various column systems, a comparative summary of traditional steel, prestressed concrete, CFST, and Concrete-Filled Fiber-Reinforced Polymer Tubes (CFFTs) is presented in Table 1. The comparison includes key factors such as construction time, labor and material cost, structural performance, durability, and environmental resistance.

**Table 1:** Comparative assessment of column systems: steel, prestressed concrete, CFST, and CFFT [27,28,29,30,31].

**Criterion**	**Steel**	**Prestressed Concrete**	**CFST**	**CFFT**
Structural performance	High	High	Very High	Very High
Construction time	Medium	Long	Short	Short
Labor cost	Medium	High	Medium	Medium
Material cost	Medium-High	High	Medium	High-Very High
Durability	Medium	Medium-High	High	Very High
Environmental resistance	Low (corrosion, humidity)	Medium	Medium-Low (outer steel exposed to environment—needs coating)	Excellent (FRP inert to chemicals, salts)
Waste/Pollution	Medium	Medium	Low (controlled production)	Very Low (precise, minimal waste)
Environmental impact	High (cutting, welding, paint fumes, noise)	Medium (pumps, post-tensioning, formwork removal)	Low (less noise, especially with prefab tubes)	Very Low (quiet installation, minimal waste, no welding or formwork)
Weight	High	High	High	Medium

## 2. Scientometric Analysis of the Bibliometric Data

**Figure 2:**
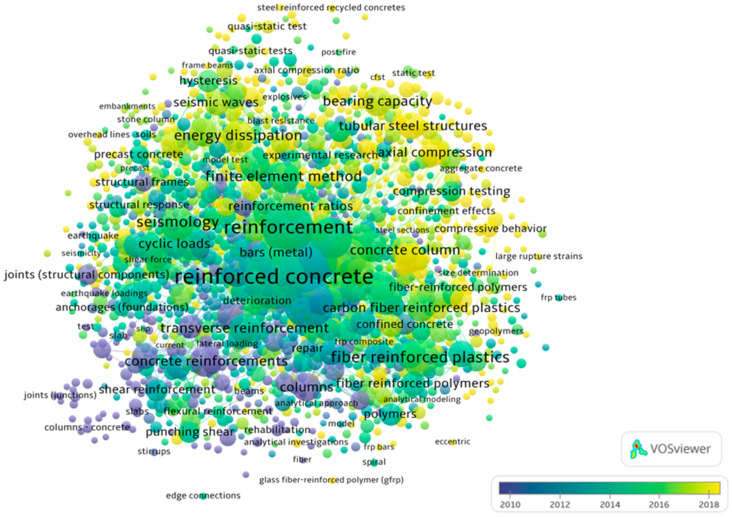
The results of the scientometric analysis by visualization of the keywords network.

Bibliometric analysis is a research method for the systematic study and evaluation of scientific output in a given field. It is based on the analysis of large bibliographic data sets (e.g., Scopus, Web of Science) such as authors, titles, keywords, citations, and affiliations. It makes it possible to determine which topics are most frequently researched, how they have evolved over the years, and which concepts are most frequently related. Bibliometric analysis provides objective and measurable information that is important for assessing the current state of knowledge and planning future research. In the case of civil engineering, a large number of papers on the reinforcement of concrete columns were identified. The Scopus database [32] was used to create a keyword map (Figure 2) in February 2025. The keywords included a combination of (“reinforcement” or “strengthening”) and (“column” or “pillar”) and “concrete”. A total of 6280 documents were identified, with a total of 20,192 keywords; the minimum number of occurrences of a word was set to 10, which made it possible to select 1533 keywords. In the context of the analysis of the keyword map generated by VOSviewer (version 1.6.20), several important trends can be observed. Clearly related topics are types of loading (e.g., cyclic load, seismic waves), suggesting that an important part of the research is focused on the analysis of the behavior of reinforced columns under load. According to the legend, the color yellow is indicative of papers published around 2018. It is evident that the use of composite materials and confined concrete is a rapidly expanding research domain.

**Figure 3:**
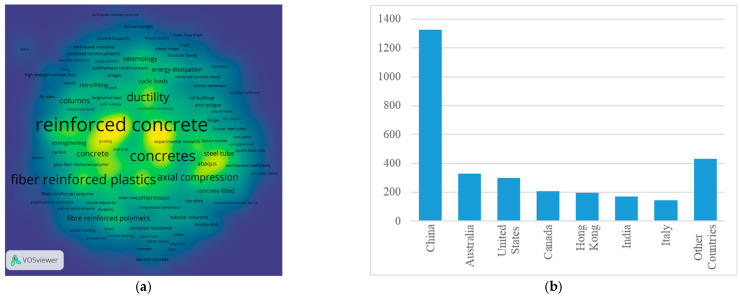
“Confined concrete column” keyword analysis: (**a**) keyword density visualization; (**b**) number of publications by country, adopted from [32].

**Figure 4:**
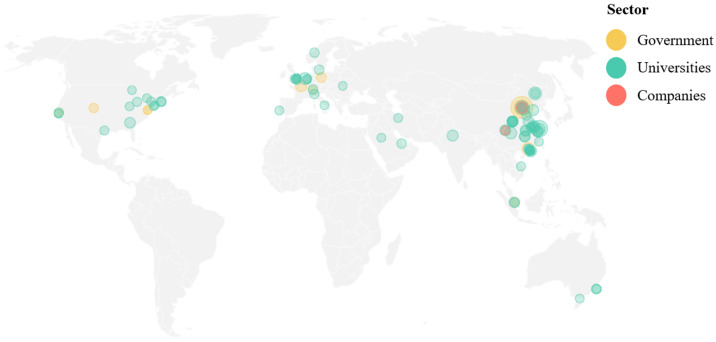
Geographic representation of institutions in the Civil and Structural Engineering sector [33].

In the subsequent phase of the bibliometric analysis, the results for the keywords “confined concrete column” were examined. The visualization of the density (Figure 3a) suggests that the primary focus of the papers is on concrete reinforcement. In addition to the common steel reinforcement, the analysis demonstrates topics related to fiber-reinforced plastics and FRP. As illustrated by Figure 3b, which displays the number of publications by country, China leads in this domain. China’s preeminence in this field is further substantiated by the ranking of academic institutions. In 2024 (Figure 4), 45 Chinese institutions were listed among the top 100 global entities in the domain of “civil and structural engineering” [33], thereby underscoring China’s leadership in both the publication of scholarly works and the development of innovative technologies pertinent to the field of construction.

**Figure 5:**
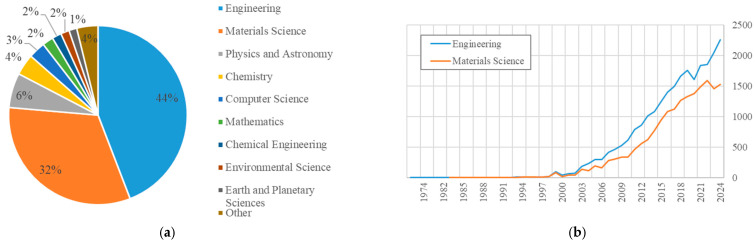
(**a**) Distribution of publications related to “Fiber Reinforced Polymer” across scientific fields; (**b**) Number of publications on “Fiber Reinforced Polymer” over the years [32].

The next step in the bibliometric analysis entailed the presentation of a distribution of articles containing the keywords “Fiber Reinforced Polymer” across various fields of study [32], as illustrated in Figure 5a, underscoring the preeminence of two specific domains: engineering and materials science. An examination of the data reveals that 44% of all publications are concentrated in the field of engineering, thereby substantiating its pivotal role in research. Materials science emerges as an equally significant domain, accounting for a substantial proportion of publications. The analysis of bibliometric data indicates a substantial increase in scientific publications related to FRP materials within engineering and materials science around 2010, as illustrated in Figure 5b, exhibiting a steady progressive trend.

As part of the bibliometric analysis conducted, four patent databases were reviewed to identify the number of patent applications related to the use of closed-section profiles (Table 2). This analysis enabled assessment of the level of innovative activity and the identification of current technological trends in the field under consideration. The results of the patent review serve as a crucial complement to the review of scientific literature, enabling a comprehensive assessment of the degree of development of technologies and areas with research and development potential.

**Table 2:** Summary of patent applications describing selected methods for reinforcing columns using closed-section profiles.

Patents	Concrete-Filled Steel Tube (CFST)	Concrete-Filled Double-Skin Steel Tubes (CFDST)	Concrete-Filled Fiber-Reinforced Polymer Tubes (CFFT)	Concrete-Filled Fiber-Reinforced Polymer-Steel Composite Tube (CFCT)	Concrete Filled Tube (CFT)
Lens [34]	42,812	3868	8818	5347	80,479
Espacenet (EPO) [35]	123,175	3493	5395	3102	208,439
PatentScope (Wipo) [36]	3330	5	7	2	4301
Google Patents [37]	125,048	124,755	70,142	41,518	125,048

## 3. Contemporary Methods of Strengthening Concrete Columns

### 3.1. Concrete-Filled Steel Tube

**Figure 6:**
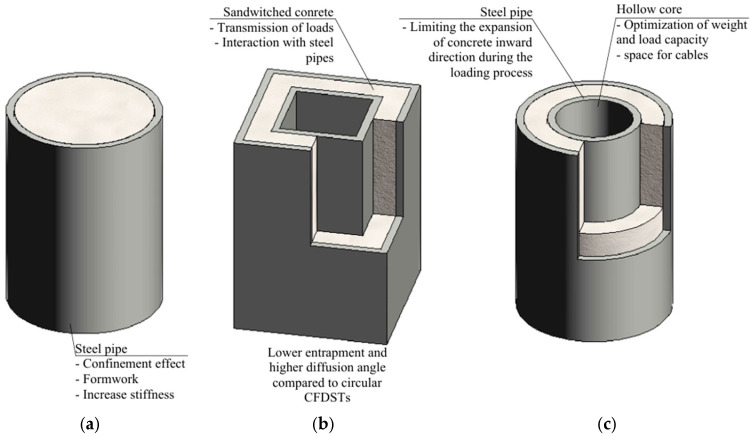
Schematic representation of: (**a**) CFST; (**b**) square CFDST; (**c**) circular CFDST.

Composite columns, which integrate materials like concrete and steel, have undergone substantial evolution in their design and applications within the construction sector. This progress has been primarily motivated by the necessity for structures with superior load-bearing capacity, enhanced fire resistance, and extended durability. The combination of concrete and steel in composite columns capitalizes on the advantageous properties of each material, resulting in significant improvements in structural performance. The confinement effect can increase compressive strength by up to 60% [38]. Figure 6 presents various configurations on CFST systems, which have gained significant attention in structural engineering due to their advantageous mechanical properties. CFSTs (Figure 6a) have become a solution in structural engineering due to their enhanced mechanical characteristics. The concrete filling improves the local stability of the steel tube, while the external steel casing enhances the concrete’s strength and durability. Shorter construction time, relatively simple implementation, and cost efficiency are additional factors that support the widespread use of CFST systems [39].

The utilization of CFST has garnered widespread adoption, attributed to their exceptional load-bearing capacity and fire-resistant characteristics. Empirical studies demonstrate that CFST columns exhibit superior mechanical properties compared to conventional steel columns, particularly under axial load conditions [40,41]. Moreover, the incorporation of ultra-high-performance concrete (UHPC) into CFST systems has been shown to significantly enhance compressive strength and longevity [42,43].

Figure 6b shows a square Concrete-Filled Double-Skin Steel Tube (CFDST), a variant investigated by Y. Yang et al. [44] under local bearing forces. Their study emphasizes the influence of geometric and material parameters, such as the width ratio, hollow ratio, and outer tube thickness. The study highlights the high bearing capacity and deformation resistance of CFDST specimens, with failure patterns consistent with those of similar composite structures. The proposed design formulae provide accurate and practical predictions for structural applications, demonstrating their relevance in engineering design. The use of an internal steel tube contributes to increased load capacity and stiffness and results in better concrete confinement [45].

Lastly, Figure 6c illustrates CFDST stub columns and beam-columns with circular hollow sections, as analyzed by Z. Tao et al. [46]. Their research focuses on key parameters, including diameter/thickness ratio, hollow section ratio, slenderness ratio, and load eccentricity. The study introduces a confinement factor to describe the composite interaction between the steel tube and sandwiched concrete, demonstrating enhanced structural behavior and ductility. Theoretical models and simplified design formulae proposed in the study show good agreement with experimental results, providing practical tools for predicting load-carrying capacities in structural applications.

Numerous experimental studies have confirmed that concrete-filled steel tube (CFST) columns outperform conventional reinforced concrete (RC) columns in terms of axial load-bearing capacity and seismic performance. The confinement provided by the steel tube significantly enhances the compressive strength, energy dissipation capacity, and ductility of the core concrete [47,48]. In CFST-RC composite frames, the integration of CFST columns with precast RC beams has proven effective in reducing buckling and instability under seismic loading, offering improved structural resilience compared to traditional RC systems [48,49]. High-strength CFST columns combined with prefabricated RC elements exhibit excellent seismic indicators, including superior displacement ductility and energy dissipation, often exceeding current seismic code requirements [49,50]. Moreover, CFST systems demonstrate rapid construction capabilities and improve fire and corrosion resistance, making them attractive for high-performance structural applications in seismic-prone regions [47].

### 3.2. Fiber Reinforced Polymer

**Figure 7:**
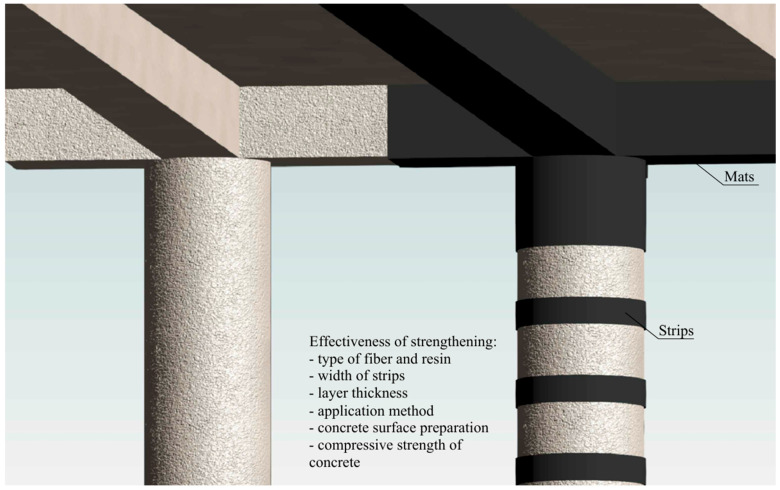
Factors affecting the effectiveness of strengthening existing concrete structures.

Fiber-reinforced polymer (FRP) composites are increasingly regarded as a viable alternative to traditional steel reinforcement, primarily due to their higher tensile strength/weight ratio and resistance to corrosion. Research conducted by Hadi et al. [51] demonstrated that the use of CFRP and glass fiber reinforced polymer (GFRP) bars enhances both axial and flexural load capacities compared to specimens without such reinforcement. CFRP reinforcement bars, compared to steel bars, exhibit a more pronounced reduction in stiffness after the initiation of cracks. Moreover, CFRP bars do not exhibit a balance point, which results from their linear elastic stress–strain behavior up to failure. In contrast, steel bars demonstrate a well-defined plastic plateau, allowing for a gradual redistribution of stresses before reaching the ultimate limit state [52]. Strengthening structures is becoming more common, since replacing damaged parts is expensive and unsustainable [53]. Figure 7 illustrates common methods for strengthening structural elements, such as the use of CFRP mats or strips, and highlights key factors that influence the effectiveness of strengthening interventions in existing concrete structures. Among these factors, the number of reinforcement layers, the type of fiber material, the geometry of application, and the condition of the original substrate all play significant roles in the final performance of the repair [54,55]. The repair parameters should take into account the characteristics of the damaged component and its effect on the stress distribution in the structure. The crucial point is to strive for an optimal match between the mechanical properties, in particular the stiffness of the repair solution used, and the parameters of the repaired component. Such an approach minimizes the risk of local stress concentration, thereby reducing the possibility of accelerated degradation and ensuring the durability of the repair [56]. The fillet of the CFRP layer contributes to the reduction of stress concentration at the edges, resulting in a delay in damage initiation and a shift in the location of cohesive failure [57].

Recycling of composite materials, especially those reinforced with carbon fiber, is becoming increasingly important due to the growing volume of waste generated from both manufacturing processes and the end-of-life phase of products such as airplanes and wind turbine blades [58]. In response to this issue, several innovative recycling methods have been developed. One approach involves mechanical processing of carbon fiber-reinforced polymer waste, which results in mixtures and refined fibers that can be added to cement-based materials. This method offers a viable solution to waste management, reduces environmental pressure, and improves resource efficiency [58]. Another promising method uses chemical recycling with meta-chloroperbenzoic acid as an oxidizing agent. The recovered carbon fibers retain nearly the same tensile strength and electrical conductivity as the original material, while also showing improved adhesion and wettability [59]. Additionally, a hybrid thermochemical recycling method, supported by a preliminary solvolysis treatment, enables the breakdown of waste at lower temperatures and allows for the recovery of fibers that maintain up to 90% of their original strength. These fibers are clean and exhibit a smoother surface with minimal residual matrix material [60]. Collectively, these strategies represent a step toward more sustainable and economically viable management of industrial composite waste.

### 3.3. Concrete-Filled Fiber-Reinforced Polymer

Concrete-filled fiber-reinforced polymer tubes (CFFTs) represent a significant advancement in structural engineering, combining the compressive strength of concrete with the tensile strength and corrosion resistance of FRP materials. This hybrid system has garnered attention for its application in various structural components, including columns, beams, and bridge piers, due to its lightweight nature and resistance to environmental degradation [61,62]. The outer FRP shell primarily bears the flexural loads, while the concrete infill enhances shear capacity, similar to the mechanics observed in sandwich panels, where the skins provide strength and the core offers shear stiffness [63]. The use of concrete filling proves more effective for thin-walled tubes or low-stiffness tubes than for thick-walled tubes or high-stiffness tubes. Table 3 outlines various CFFT column configurations along with key experimental findings regarding their structural performance.

**Table 3:** Experimental findings on selected CFFT structural systems.

Construction	Reference	The Main Conclusions
Square concrete-filled double skin steel tube (CFDST)	Y. Yang et al. [44]	Proposed design methods providing accurate and safe predictions
Compressive behavior of CFFTs (HSCFFTs and UHSCFFTs)	T. Vincent and T. Ozbakkaloglu [64]	CFFTs filled with high- and ultra-high-strength concrete exhibit highly ductile behavior and significant strength gains after initial peak load; The FRP tube manufacturing method strongly affects the compressive performance, with automated filament winding producing better results compared to manual wet lay-up
Seismic behavior of square HSC-filled FRP tubes (HSCFFTs)	Y. Idris and T. Ozbakkaloglu [65]	Square HSCFFT columns demonstrate high inelastic deformation capacities under simulated seismic loading; Column deformability decreases with higher axial load levels and concrete strength, but increased confinement can mitigate this effect; Increasing the corner radius improves drift capacity up to a threshold, beyond which no further improvements are observed
Flexural behavior of concrete-filled FRP-steel composite circular tubes	Y. Wei et al. [66]	FRP enhances ultimate bearing capacity, introduces a hardening stage after steel yielding, and prevents “zero stiffness.”; The combination of various FRP types mitigates fracture failure by enabling sequential fiber rupture and altering failure modes

The study [62] examines the behavior of circular concrete-filled fiber-reinforced polymer-steel composite tube (CFCT) columns under axial compression. The study demonstrated that fiber-reinforced polymer strengthening effectively enhances compressive strength, improves lateral deformation stability, and delays local buckling of the steel tube, while the developed theoretical model accurately predicts the load-bearing capacity of these composite columns, considering their composite action. The FRP tube serves not only as a formwork but also as a strengthening mechanism, confining the concrete and improving its load-carrying capacity and ductility [64]. This confinement effect is crucial, as it allows for enhanced performance under axial loads, which is particularly beneficial in seismic-prone areas [65]. The application of a hybrid approach involving the implementation of an internal and external FRP layer in a steel tube filled with concrete serves as an effective barrier against chloride ion penetration [67,68]. This solution contributes to enhancing the durability of structures operating in aggressive marine environments. In addition, the use of FRP composites as the outer shell and protective layer of the inner steel tube allows the use of concrete containing sea-sand, which expands the range of available construction materials and can contribute to cost optimization and sustainable use of natural resources [69]. The use of added CFRP strengthening affects the axial compressive strength and stiffness of the columns. The presence of CFRP positively influences the failure mechanism of filled square steel tube columns by reducing the strain concentration in both the steel tube and the concrete [70]. However, the effectiveness of the CFRP confinement is higher for circular columns than for square columns [71]. The study [72] explores the behavior of UHPC-filled FRP tubular columns under combined axial and flexural loading. The results highlight that FRP confinement improves ductility, energy absorption, and deformation capacity compared to unconfined UHPC columns, particularly under eccentric loading. Additionally, the proposed analytical model provides reliable predictions of structural performance, aligning well with experimental findings.

Recent studies have also highlighted the beneficial effects of fiber-reinforced polymer composites on the dynamic performance of concrete columns. CFRP mats significantly increase axial impact resistance, improve fracture toughness, and enhance the structure’s capacity to dissipate energy under sudden loads [73]. Similarly, GFRP tubes contribute to better damage resistance, energy absorption, and reduced residual deformations in impact-loaded concrete columns [74]. Increasing the thickness of GFRP layers can further improve energy dissipation and reduce maximum displacement, though it has a limited influence on peak impact force [75]. Unconfined FRP columns demonstrate insufficient energy dissipation, making them less suitable for seismic applications, whereas hybrid systems (HCFFTs) incorporating steel fibers into the FRP shell exhibit enhanced ductility and energy absorption—qualities essential for bridge columns in seismic zones [76].

FRP tubes exhibit poor fire resistance and higher flammability, which has been repeatedly studied [72,77]. In particular, the softening of the epoxy resin, typically occurring at temperatures between 60 °C and 140 °C, leads to the degradation of the bond between the FRP and the concrete surface and initiates internal spalling due to trapped moisture [78]. Early ignition of CFRP layers beneath intumescent coatings has been observed within the first 15 min of fire exposure, causing a rapid increase in surface temperature and contributing to loss of integrity [78]. On the other hand, a study [79] analyzed the effect of fire exposure on the structural properties of FRP-encased concrete columns. Exposure to fire significantly reduces the load-bearing capacity of the columns, and FRP tubes almost completely lose their ability to effectively restrict. This process is due to the combustion and charring of the resin, as confirmed by microstructural analysis and observations of the resulting damage. The study shows that the use of a fire-resistant additive is effective in improving the fire resistance, limiting the temperature rise, preserving the structural properties and load-bearing capacity of the encased cylinders, and increasing their deformability.

Despite these thermal limitations, FRP materials have shown excellent durability in chemically aggressive environments, such as seawater and crude oil. Experimental data indicate that CFRP confinement improves both strength and ductility of concrete columns exposed to such conditions. Compared to unconfined specimens, CFRP-wrapped columns exhibited an increase in load-bearing capacity by approximately 61–64% under monotonic loading in seawater and up to 73.7% under cyclic loading; in crude oil, improvements ranged from 45.6% to 59.2% [80]. In addition, CFRP wrapping has been shown to effectively reduce microcracking in concrete columns exposed to environmental stressors [81].

The performance of FRP-confined concrete columns under freeze-thaw cycles (FTCs) exhibits a notable dependence on the type of reinforcement, column geometry, and environmental conditions. Studies have shown that although the overall load-bearing capacity tends to decrease with an increasing number of FTCs, FRP confinement can mitigate the extent of deterioration. For instance, circular concrete specimens confined with CFRP tubes demonstrated significantly higher frost resistance compared to square counterparts, with a maximum average strength loss of 27.6% after 60 FTCs for 100 mm high columns, and as low as 10.17% for taller specimens [82]. Similarly, partially wrapped RC columns using CFRP or GFRP showed that tensile strength of both materials was reduced post-FTC, with GFRP being more vulnerable due to higher water absorption. Despite this, CFRP-confined columns retained superior compressive performance under eccentric loads [83]. In addition, concrete-filled GFRP tubes exhibited a marked reduction in both ultimate strength and initial stiffness after FTC exposure, especially in square columns, although increased column height appeared to slightly improve resistance to freezing damage [84]. While the bond strength between externally bonded CFRP laminates and concrete showed minimal degradation and even slight improvements in some freeze-thaw environments, this did not directly translate into enhanced structural capacity of whole columns [85].

**Figure 8:**
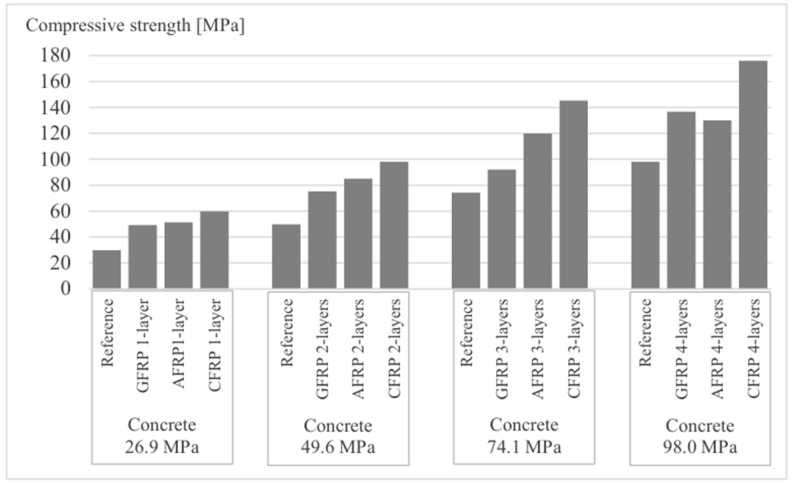
Effect of fiber type and number of layers on the effectiveness of concrete column strengthening, adapted from [99].

A review of the literature shows that researchers have extensively studied the effect of different types of fibers on the strengthening of concrete columns. Among the materials studied, the best strengthening effect is obtained with CFRP fibers, which have exceptional strength and durability. Table 4 compares the effect of different FRP materials on strengthening effectiveness, considering AFRP, CFRP, GFRP, and BFRP. The results show that CFRP fibers provide the best results in improving the strength and durability of concrete columns, which is prompted by the best properties (Table 5). In addition, Figure 8 illustrates the nonlinearity of the effectiveness as the number of layers increases.

**Table 4:** Influence of FRP composite type on concrete behavior.

FRP Type and Layer	Layer Plain	Slenderness	$\mathrm{Compressive}\;\mathrm{Strength}\;\mathrm{of}\;\mathrm{Unconfined}\;\mathrm{Concrete}\;f_{co}$ (MPa)	$\mathrm{Compressive}\;\mathrm{Strength}\;\mathrm{of}\;\mathrm{Strengthened}\;\mathrm{Concrete}\;f_{cu}$ (MPa)	$\frac{f_{cu}}{f_{co}}$	Ultimate Strain (%)	Reference
AFRP	1	2	35.5	66.3	1.87	1.90	[86]
			39.2	60.0	1.53	2.24	[87]
PEN FRP ^1^				52.6	1.34	3.68	
PET-900 FRP ^2^				48.0	1.22	4.89	
PET-600 FRP ^2^			32.5	41.8	1.29	4.77	
GFRP			35.2	63.8	1.81	1.97	[88]
CFRP				71.6	2.03	2.21	
BFRP	2			44.1	1.25	1.15	
CFRP + GFRP	1 + 1		19.70	65.56	3.32	2.40	[89]
GFRP + CFRP	1 + 1			44.34	2.25	1.76	
CFRP	1		51.1	70.0	1.37	6.90	[90]
			36.0	41.8	1.16	2.90	[91]
HM CFRP			36.3	45.2	1.25	2.77	
GFRP			33.1	42.0	1.27	1.29	[92]
CFRP			37.7	49.4	1.31	0.90	
JFRP ^3^ + BFRP	2 + 2		22.8	38.5	1.69	1.80	[93]
BFRP	2			31.0	1.36	1.60	
JFRP ^3^				27.7	1.22	1.48	

^1^ Polyethylene naphthalate; ^2^ Polyethylene terephthalate; materials of different thicknesses, i.e., PET-900 1.262 mm and PET-600 0.841 mm; ^3^ Jute fiber reinforced polymer.

**Table 5:** Fiber properties, adapted from [86,94,95,96,97,98].

**Type of Fiber**	**Elastic Modulus (GPa)**	**Ultimate Tensile Strain (%)**	**Tensile Strength (MPa)**	**Density (kg/m^3^)**
Aramid	70–179	2.50	2900–3450	1440–1470
Carbon	230–240	1.55	3700–3900	1800
HM Carbon	390–640	0.40	2600–4900	2000
Glass	65–72	1.93–2.12	1700–3400	2460–2580
Basalt	80–100	2.25–3.1	3300–4500	2600–2800

#### 3.3.1. Size Effects in FRP-Wrapped Concrete Columns

The effect of scale in concrete columns strengthened with FRP composites remains a subject of intensive research, as it can significantly affect their strengthening mechanisms and the final strength parameters of the structure. As indicated in the studies [100,101,102], for CFRP-confined columns, under identical lateral confining stress, the strength gain, normalized ultimate strain, and stress–strain relationship do not show an effect size. In contrast to these results, Wang et al. [103] showed that specimen size has an effect on the transitional and ultimate strength ratios, especially at lower confinement ratios. However, under higher confinement ratio conditions, the scale effect becomes less significant. These results are reflected in the data presented in Table 6, where some variation in the $F_{cu}/F_{c0}\;$ ratio is observed for samples of different sizes. Moreover, studies on the effect of the slenderness of FRP-confined columns show that the results for columns with different H/D ratios show no significant difference (Table 7).

**Table 6:** Summary results of research on the effect of specimen size on the compressive strength of FRP-strengthened concrete elements.

Fiber Type	Diameter (D) and Height (mm)	$\mathrm{Thickness}\;t_{f}$ (mm)	$\mathrm{Confinement}\;\mathrm{Ratio}\;\frac{4t_{f}}{D}$	Compressive Strength of Unconfined Concrete (MPa)	Compressive Strength of Strengthened Concrete (MPa)	$\frac{F_{cu}}{F_{c0}}$	References
CFRP	100 × 200	0.167	0.00668	25.9	64.6	2.49	[100]
	200 × 400	0.334		22.7	64.9	2.86	
	300 × 600	0.501		24.5	60.5	2.47	
AFRP	70 × 100	0.0572	0.0033	29.4	41.8	1.45	[103]
		0.0953	0.0054		49.64	1.72	
		0.1907	0.0109		86.07	2.99	
	105 × 315	0.0715	0.0027	28.8	41.2	1.43	
		0.143	0.0054		47.77	1.66	
		0.286	0.0109		87.42	3.04	
	194 × 582	0.1430	0.0029	24.0	33.84	1.18	
		0.2860	0.0059		43.9	1.52	
		0.5720	0.0118		80.86	2.81	
CFRP	50 × 100	1	0.04	53.8	146.2	2.72	[101]
	100 × 200			49.1	94.5	1.92	
		2	0.08		146	2.97	
	150 × 300			41.1	111.5	2.71	
		1	0.04		76.4	1.86	

**Table 7:** Effect of slenderness on compressive strength of FRP-strengthened circular columns.

**Fiber Type**	**Height (mm)**	**Diameter (mm)**	**Slenderness Ratio**	**Number of Layers of** **Composite**	**Compressive Strength of Unconfined Concrete (MPa)**	**Compressive Strength of Strengthened Concrete (MPa)**	$\frac{F_{cu}}{F_{c0}}$	**References**
GFRP	300	150	2	2	30.6	89.5	2.92	[104]
	450		3			91.2	2.98	
	600		4			85.8	2.80	
	750		5		30.4	87.6	2.89	
				3		128.1	4.22	
		250	3	2	31.2	55.8	1.79	
CFRP	600	150	4	2	28.2	57.4	2.04	[94]
HM CFRP						65.6	2.33	
GFRP						35.8	1.27	
CFRP	304	152	2	1	36	64	1.78	[105]
	912		6	2	37	64	1.73	
GFRP	304		2	3	36	90	2.50	
	902		6		37	87	2.35	

#### 3.3.2. Relationship Between CFRP Strengthening Effectiveness and Concrete Strength Class

There are numerous scientific studies in the literature on the effectiveness of strengthening concrete columns with FRP composite sheets. Table 8 presents the results of studies in which the variable parameters were the compressive strength of the unreinforced concrete and the number of CFRP layers, while the type of fiber, matrix, and specimen dimensions remained unchanged. The analysis of the data collected indicates that the effectiveness of the reinforcement is more pronounced in concrete with lower compressive strength. In terms of deformability, a more favorable improvement is also observed for concrete with lower strength classes. These results suggest that lower-strength concrete not only experiences a greater relative strength increase but also undergoes a more significant improvement in its deformability after CFRP reinforcement. In addition, an analysis of the effect of the number of CFRP layers showed that an increased number of layers leads to an improvement in both the compressive strength and deformability of the reinforced elements. However, this effect is not linear—the increases in strength and deformability decrease with each additional layer, suggesting the existence of an optimal number of layers for a given concrete type and composite material. Increased porosity in recycled aggregates results in lower final stiffness due to more crack planes compared to concrete containing stone aggregate.

#### 3.3.3. Failure Modes of FRP Strengthened Columns

**Figure 9:**
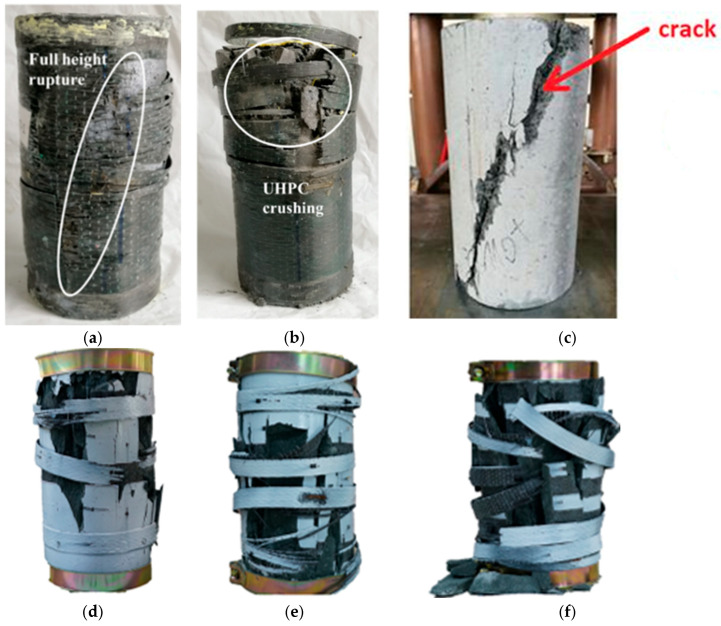
Concrete column failure model with: (**a**) single-layer strengthened CFRP [111]; (**b**) three-layer strengthened CFRP [111]; (**c**) CFCR ring [113]; (**d**–**f**) partial wrapping 3-, 4-, 5-strips [112].

The strengthening of concrete columns by wrapping them with FRP mats significantly affects their failure, leading to a sudden failure around the mid-height of the element [111]. In the case of smaller specimens (height 10 cm), cracks develop mainly in the circumferential direction, whereas for larger specimens (height 20 cm), damage initiation occurs along the fiber winding direction. Moreover, an increase in the number of FRP layers results in the accumulation of greater strain energy, leading to multi-segment concrete failure (Figure 9b). In contrast, for single-layer strengthening, a single inclined crack is the dominant failure mode (Figure 9a). A slightly different failure mechanism is observed in concrete columns that are partially strengthened with FRP strips [112]. In the unwrapped concrete areas, vertical cracks appear, similar to those found in non-strengthened elements, whereas in the FRP-confined zones, diagonal shear cracks dominate due to the triaxial stress state (axial compression and lateral confining pressure). Consequently, vertical and diagonal cracks gradually merge, forming a primary crack that initiates failure (Figure 9d–f). Unlike CFRP-strengthened columns, elements strengthened using the CFCR (carbon fiber coated reinforcement) method [113] do not exhibit sudden failure. In such columns, a dominant vertical crack extending along the entire height of the specimen is observed, originating at the interface between the concrete and the CFCR ring (Figure 9c). At later loading stages, multiple secondary cracks appear, eventually leading to a loss of load-bearing capacity. The structural integrity of these specimens is attributed to the presence of dispersed steel reinforcement in the concrete.

#### 3.3.4. Load-Bearing Capacity Estimation

A number of models have been developed for the purpose of predicting the behavior of concrete in compression, especially in the context of confinement by FRP, and selected models are summarized in Table 9. In [114], the authors presented a simple empirical design-oriented model for predicting the strength of strengthened concrete members with confinement by FRP. The work of [115], on the other hand, focuses on a new stress–strain model for FRP-confined concrete that is simple in its design use, yet takes into account important behavioral characteristics and reduces to existing standard models for unconfined concrete. Additionally, a standardized model for the ultimate deformation of FRP-confinement concrete has been proposed, taking into account different types of strengthening material. In [100], an analytical-type model was developed for stress–strain and strength for CFRP-confinement concrete columns. This model was compared with a comprehensive database of strength models (integral absolute error rate). In addition, source [116] presents a novel design-oriented model for the prediction of the ultimate state of confined FRP concrete. This model is based on an extensive experimental database and includes expressions for ultimate axial strain and related coefficients. In [117], a novel model for the ultimate strain of FRP-trapped concrete and an empirical formula for the ultimate Poisson’s ratio were proposed, along with equations for predicting maximum strength and ultimate strain for concrete with strain-softening response. A work [118] describes a general stress–strain model applicable to both steel- and FRP-confined concrete, which is an extension of the Popovics model and provides a unified approach to modeling the behavior of concrete confined with different materials, along with models for evaluating the peak and ultimate points of the stress–strain curve. In contrast, in a paper by Dai et al. [87], the authors proposed a modification of the analytical model [119] that more precisely predicts the behavior of FRP-strengthened concrete with large rupture strain (LRS FRP).

**Table 9:** Models for the confinement of axially loaded confined concrete in circular sections.

Type of Confinement	Ultimate Compressive Strength	Ultimate Axial Strain	Reference
FRP	$\frac{f_{cc}}{f_{co}}=1+0.5\left(\frac{\rho_{f}E_{frp}}{f_{co}}\right)(-\frac{0.4142E_{f}10^{-6}}{E_{f\mu }}+0.0248)$	$\varepsilon_{je}k_{1}=2\left(-\frac{0.4142E_{f}10^{-6}}{E_{f\mu }}+0.0248\right)$	[114]
FRP	$\frac{f_{cc}^{\prime }}{f_{co}}=1+3.3\frac{f_{l,a}}{f_{co}^{\prime }}$, $f_{l,a}=\frac{2E_{frp}t\varepsilon_{h,rup}}{d}$	$\frac{\varepsilon_{cu}}{\varepsilon_{co}}=1.75+12\left(\frac{E_{frp}t}{E_{seco}\;R}\right){\left(\frac{\varepsilon_{h,rup}}{\varepsilon_{co}}\right)}^{0.45}$	[115]
FRP	$\frac{f_{cc}^{\prime }}{f_{co}^{\;⠀}}=1+(2.61-{0.01f}_{co}^{\prime })\frac{f_{l}}{f_{co}^{\prime }}$	$\varepsilon_{l}^{\;⠀}=-0.2\varepsilon_{c}^{\;⠀}-6\times 10^{-4}\times {\left(\left(\varepsilon_{co}-0.001\right)\times 10^{3}\right)}^{0.00725f_{c}^{\prime }+1.355}$	[100]
FRP	$f_{cc}^{\prime }=1+0.0058\frac{k_{1}}{f_{co}^{\prime }}+\frac{k_{1}\left(f_{l,a}-f_{lo}\right)}{f_{co}^{\prime }}$	$\varepsilon_{cu}=c_{2}+k_{2}{\left(\frac{K_{l}}{f_{co}^{\;⠀}}\right)}^{0.9}\varepsilon_{h,rup}^{1.35}$	[116]
FRP	$f_{cc}^{\prime }=f_{co}^{\prime }+k_{1}f_{lu}$	$\varepsilon_{cc}^{\prime }=\frac{\varepsilon_{fu}}{\nu_{u}}$, $\nu_{u}=0.56k_{1}{\left(\frac{f_{1}}{f_{co}^{\;⠀}}\right)}^{-0.66}$	[117]
FRP	$\frac{f_{cc}}{f_{co}}=1+0.0015\frac{E_{l}}{f_{co}^{0.5}}$	$\frac{\varepsilon_{cc}}{\varepsilon_{co}}=1+0.003\frac{E_{l}}{f_{co}^{0.5}}$	[118]
LRS FRP	$f\left(\frac{\varepsilon_{l}}{\varepsilon_{co}}\right)=1.024{\left(\frac{\varepsilon_{l}}{\varepsilon_{co}}\right)}^{0.35}+0.089\left(\frac{\varepsilon_{l}}{\varepsilon_{co}}\right)$	$\frac{\varepsilon_{c}}{\varepsilon_{co}}=\left(1+8\frac{\sigma_{l}}{f_{co}^{\;⠀}}\right)\left[1.024{\left(\frac{\varepsilon_{l}}{\varepsilon_{co}}\right)}^{0.35}+0.089\left(\frac{\varepsilon_{l}}{\varepsilon_{co}}\right)\right]$	[87]

### 3.4. Perforated FRP Tubes

The study by Wang et al. [120] considered the influence of hole diameter and perforation pattern, as well as tube diameter, on load-bearing capacity and stiffness. The findings indicate that perforation of the GFRP tube leads to a significant reduction in both load-bearing capacity and stiffness, with the distribution of holes affecting the performance of the perforated tube; axial perforation proved to be more favorable than diagonal perforation. Furthermore, another conclusion is that the hole diameter also significantly affects the structural properties. In the study by Taheri-Behrooz et al. [121], it was stated that increasing the hole diameter and spacing within a reasonable range would not cause significant changes in structural behavior. However, in this research, relatively small holes were analyzed in comparison to the tube diameter, which may influence the generalizability of the results.

Another paper by Wang et al. [122] examined the impact of different GFRP tube strengthening variants on concrete columns—intact GFRP tubes, intact GFRP tubes with a polymer mesh, and perforated GFRP tubes. The results of the study demonstrated that columns strengthened with an intact GFRP tube combined with a polymer mesh exhibited higher load-bearing capacity and better crack control compared to those strengthened with perforated tubes, where the presence of holes weakened the structure.

**Figure 10:**
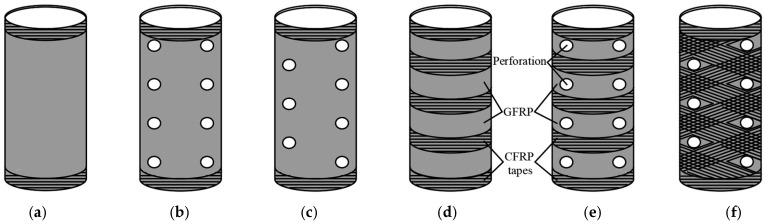
GFRP strengthening: (**a**) unperforated; (**b**) with axial perforation; (**c**) with diagonal perforation; (**d**) unperforated strengthened horizontally with CFRP tapes; (**e**) with axial perforation strengthened horizontally with CFRP tapes; (**f**) with diagonal perforation cross-strengthened with CFRP tapes.

In the studies [123], an analysis was performed on the strengthening of concrete columns with perforated GFRP tubes. The perforations in the composite material enabled the integration of the concrete core with the protective layer, with the aim of limiting premature spalling of the concrete cover. To prevent premature cracking of the GFRP tubes and enhance their circumferential tensile strength, additional strengthening was applied in the form of two layers of CFRP sheets wrapped around the tube surface. All specimens were strengthened with CFRP tapes along the upper and lower edges. For three types of samples, additional reinforcing tapes were applied in different configurations: in a cross pattern around the holes, in a horizontal pattern between the holes, and at equal intervals for the sample without perforations (Figure 10). The study’s findings indicated that the application of additional strengthening did not result in a significant improvement in the ultimate load capacity or the associated deformations of the columns. It was observed that all samples failed in a brittle manner. In the case of GFRP tubes, failure occurred due to stress concentration at their ends, whereas perforated GFRP tubes were damaged due to cracking around the holes. Specifically, in samples with axial holes, cracks propagated longitudinally around the perforations. In contrast, in samples where diagonal perforations in GFRP tubes were combined with additional strengthening using CFRP tapes, failure initiated from cracks around the holes. Meanwhile, columns without CFRP tapes failed due to longitudinal cracking in the central part of three adjacent holes.

It is clear that the propagation of damage in composite tubes is initiated in areas weakened by perforation, and hole size is crucial [124,125,126]. Based on the study [127], the authors concluded that an increase in the number of perforations around the transverse direction of square CFRP tubular profiles, both on adjacent and opposite surfaces, has a marginal effect on peak load. Similarly, the geometry of the perforations plays a limited role in shaping the value of the critical load, with square perforations exhibiting only a 7% higher load-bearing capacity compared to circular perforations. However, significant differences arise in the context of specific energy absorption capability. Specifically, circular holes exhibit an energy absorption capacity of less than 70% in comparison to samples with square holes. These differences stem from distinct mechanisms of structural degradation under loading conditions. Jiang and et al. [128] conducted studies to improve the energy absorption capacity of perforated CFRP tubes by applying patches of the same material. It was demonstrated that strengthening the area around the hole results in stress redistribution, which reduces stress concentration and transfers stresses to the tube ends, thereby delaying the initiation of cracks in the perforation region. The implementation of a patch around the hole influences the structural degradation mechanism—instead of a sudden fracture in the central part, a more controlled and gradual crushing is observed. However, the effectiveness of this method is limited by the bond strength, which defines the upper limit for increasing the number of strengthening layers. Furthermore, the analysis has shown that the application of patches on both sides of the tube leads to a more effective reduction of the stress concentration compared to the use of multilayer patches on one side.

### 3.5. Strengthening of Concrete Columns by Strips

**Figure 11:**
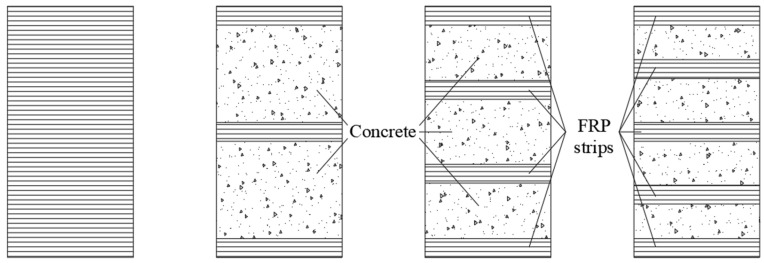
Strengthening: fully; partially (3-, 4-, 5-strips).

Existing concrete columns can be strengthened by wrapping them with an FRP composite jacket in the circumferential direction, either fully or partially (Figure 11), which helps reduce costs. Partial confinement significantly enhances both strength and axial deformation capacity, while the failure mechanism remains similar to that of fully wrapped FRP columns, characterized by the rupture of composite strips. However, increasing the spacing between FRP strips may lead to concrete failure in the unconfined regions.

**Figure 12:**
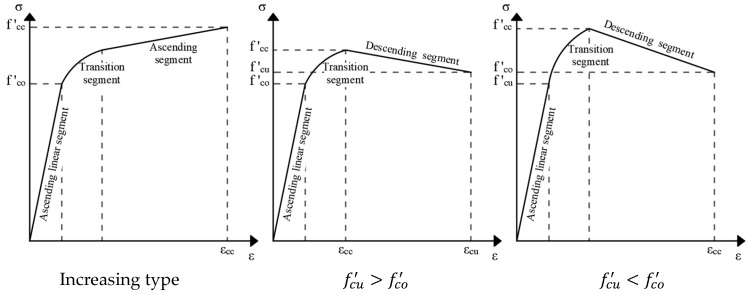
Typical stress–strain curves for FRP-confined concrete in partially wrapped concrete columns, adapted from [129].

The stress–strain analysis of FRP-strengthened columns reveals a distinct pattern consisting of three segments: an initial ascending linear segment, a transition segment, and a final ascending or descending segment (Figure 12) [129], depending on the applied strengthening configuration. The slope of the third segment increases with the width of the FRP strips, whereas the effect of strip thickness on this parameter is negligible. Both the width and thickness of FRP strips influence the ultimate strain of the concrete; however, they remain independent of the spacing between adjacent composite strips. Additionally, in partially wrapped FRP columns, an uneven distribution of compressive forces has been observed, leading to the so-called “arching effect” [130].

In the study [131], the behavior of slender columns strengthened with a hybrid composite system was analyzed. Longitudinal CFRP strips were used to enhance flexural stiffness and buckling resistance, while the columns were further strengthened with GFRP sheets. Unlike in smaller columns, this approach in slender columns resulted in increased axial load capacity, flexural stiffness, and lateral displacement capacity under peak loading conditions.

The use of rubber additives in concrete contributes to sustainable development; however, it also leads to a reduction in structural load-bearing capacity. The study presented in [132] demonstrated that the adverse effects of decreased compressive strength and limited deformation capacity can be mitigated by encasing the concrete column in a PVC tube and strengthening it with FRP strips. This approach not only enhances the mechanical properties of concrete but also reduces the consumption of composite materials by nearly 50%.

## 4. FRP Textiles and Their Application in Concrete Structures

### 4.1. Orientation of Fibers

The study [133,134,135,136] showed that the orientation of the fibers in the FRP jacket plays a critical role in the axial capacity of FRP-confined concrete. The highest performance was achieved when the fibers were oriented in the hoop direction, which provided effective confinement and significantly improved the compressive behavior of the concrete. As the fiber angle deviated from the hoop direction, the efficiency of the fibers decreased significantly. In addition, the method of confinement, whether by CFFT tubes or FRP wrapping, did not significantly affect the axial stress–strain behavior, although minor differences in ultimate strain and peak stress were observed. Similar conclusions can be found in [137], where the effect of fiber orientation on mechanical properties was investigated. The results showed that systems oriented at 0° had the best mechanical performance, while systems with other orientations (15–75°) showed a significant reduction in strength, such as 18.9–50.2% for shear strength and 32.4–57.8% for tensile strength.

**Figure 13:**
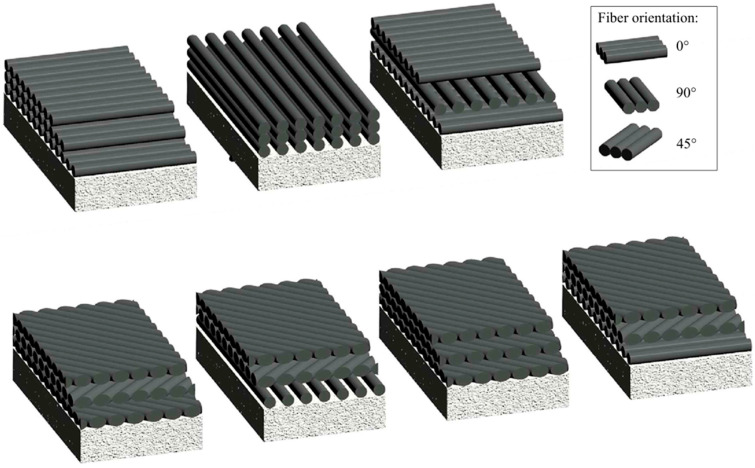
Configurations of fiber orientations in multilayer CFRP laminates with varying angular arrangements, adapted from [143].

Fiber orientation (Figure 13) also affects stress–strain behavior. A study by P. Sadeghian et al. [138] investigated the effect of fiber orientation (0°, 45°, and 90°) and number of plies (1–4 plies). It was shown that longitudinal (0°) and transverse (90°) configurations exhibited linear elastic behavior ending in brittle fracture, while angular (45°) configurations exhibited nonlinear behavior with high ductility. For mixed configurations (0° and 45°), a nonlinear stress–strain curve with maximum strength was observed, followed by ductile failure with nonlinear softening. On the other hand, in a study by Piekarczyk et al. [139], the results show that the combination of unidirectional laminates (1D) and 45°/45°, applied to the surface of the specimens, makes it possible to obtain concrete columns with high compressive strength and increased ductility. The selection of fiber orientation in composites should be determined by the intended functional properties. When ductility is the primary requirement, the ±45° arrangement allows for greater deformation before failure. Conversely, if the design aims to maximize mechanical strength under both static and dynamic loads, a multidirectional layout [±45°, 0°] is recommended, as it provides a more favorable stress distribution [140]. Laminate design is based on the analysis of design charts (carpet plots), which illustrate the influence of the proportion of layers with different orientations on the strength and other material properties [141]. This method enables the rational selection of layer sequences and proportions to meet specific design criteria. The stacking sequence in a laminate also influences the magnitude of thermal stresses that develop during cooling after curing. Differences in the coefficients of thermal expansion between the fibers and the matrix, as well as the orientation of the layers, can generate stresses that lead to matrix microcracking, which adversely affects the mechanical properties of the composite [142].

Over the years, research on FRP composites has focused on finding more sustainable solutions. One direction is to blend fiber types. In research [144], carbon–glass hybrid composites were used, resulting in improved tensile and flexural strengths compared to pure FRP. In addition, there was an increase in tensile elongation (2.84%) compared to ready-mixed CFRP (1.36%). This solution makes it possible to achieve mechanical properties similar to CFRP at a lower cost. Another solution to minimize the carbon footprint and increase biodegradability is the use of synthetic and natural fibers. A study [145] analyzed the effect of adding natural fibers (jute, flax, coconut, and hair) to GFRP. The use of jute fibers improved stiffness, and tensile strength by 36%, compared to pure GFRP. On the other hand, an improvement in flexural strength was achieved with glass–flax fiber combination.

### 4.2. Lamination Process

**Figure 14:**
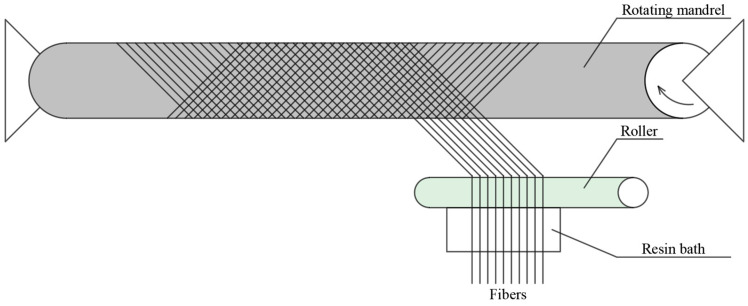
Filament winding process.

The method of manufacturing FRP composites has a significant impact on their mechanical properties, functionality, and cost effectiveness. The choice of technology depends on several factors, such as production volume and labor costs. For example, the pultrusion process produces a high fiber/volume ratio, resulting in increased strength and stiffness of the material. Hand lay-up, on the other hand, offers greater design flexibility and allows for specific lay-ups. Filament winding (Figure 14), often used in cylindrical structures, provides excellent strength along the direction of fiber placement [146].

The wet lay-up method involves the manual application of resin to the reinforcing material directly on the surface of a mold. This process does not require the use of additional thermal or pressure-based treatments [147]. This technique enables the fabrication of large components with complex geometries without the necessity for expensive molds or specialized equipment typically required in processes such as hot pressing or injection molding. In contrast to other composite manufacturing methods, the wet lay-up process does not provide precise control over the thickness of the layers or the fiber/matrix volume ratio [148]. The impregnation of the reinforcing fabric is typically carried out using rollers, which enhances fiber wetting and reduces air pockets that may remain trapped within the fiber bundles [149]. Due to limited repeatability and the challenges associated with achieving uniform mechanical properties, this method is not recommended for mass production [150]. The wet lay-up method is widely applied in the repair and maintenance of large-scale composite structures [151,152,153].

**Figure 15:**
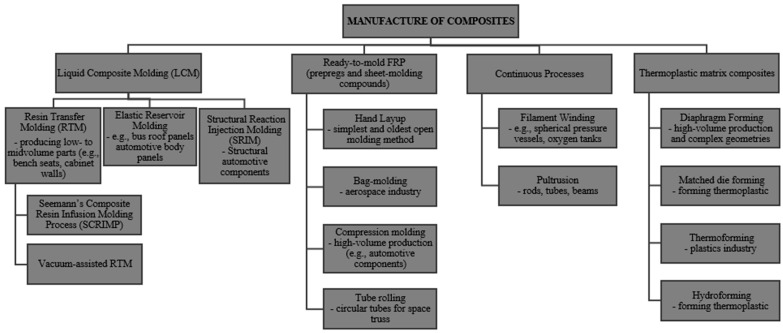
Composite manufacturing methods and their applications, adapted from [142].

One of the key limitations of using this technique in enclosed manufacturing environments is the emission of volatile resin vapors during processing with an open mold. This issue is mitigated in the Vacuum Infusion method, where a vacuum is applied prior to resin introduction, and the resin is subsequently drawn into the laminate [154]. An advancement of this technology is the Double Vacuum Bagging method, in which the outer vacuum bag compacts the composite, while the inner bag facilitates the removal of volatiles, trapped air, and moisture, thereby supporting more uniform fiber impregnation [155]. Other known composite manufacturing methods are shown in Figure 15.

As mentioned in Section 3.2, composite materials are widely used to strengthen damaged concrete elements through the technique of Externally Bonded Reinforcement (EBR) or Near Surface Mounted (NSM) systems.

The wet lay-up FRP technique reinforces deteriorated concrete by directly applying fiber-reinforced polymer (FRP) onto a properly prepared surface. After surface preparation, a two-component epoxy resin is applied to both the concrete substrate and the FRP sheets, which are then bonded to the surface and left to fully cure. In the dry lay-up method, the first step involves spreading epoxy resin over the concrete surface, after which the FRP sheets are applied. These sheets are then pressed and laminated along the fiber direction using a roller [96], which facilitates even resin impregnation of the fibers and the elimination of air bubbles.

In the NSM technique, grooves are cut into the concrete cover of the reinforced concrete element, into which FRP rods or strips are inserted and bonded using a suitable filler material [156,157,158]. The advantage of this method lies in the reduced risk of debonding, enhanced protection of the FRP against external factors, and a higher increase in the strength of the strengthened component [158,159,160,161,162,163]. Additionally, NSM reduces the risk of damage caused by vandalism, mechanical impact, and material aging while having little effect on the appearance of the structural element [164].

The effectiveness of both EBR and NSM techniques strongly depends on the quality of the bond, which is influenced by factors such as the roughness of the concrete surface, groove stresses, the mechanical properties of the FRP material, and the type of adhesive used [165]. Regardless of the chosen method, both approaches effectively strengthen concrete elements, significantly improving their load-bearing capacity, durability, and service life.

### 4.3. Epoxy Resins

Polymer resins can be divided into thermoplastic and thermosetting, the main difference being the polymerization process. One of the most commonly used thermosetting resins is polyester, which is characterized by its low cost, initial liquid consistency, and low viscosity, allowing for a wide range of applications. It also has high chemical and environmental resistance. However, matrix shrinkage occurs during curing, which can lead to internal stresses. It should also be noted that polyester resin contains styrene, which is difficult to recycle [79].

Vinylesters, although showing similarities to polyesters (processing and curing method), have higher flexibility and resistance to cracking. In addition, they show resistance to strong acids and alkalis, making them suitable for use in demanding environments. Due to the presence of hydroxyl groups, vinylesters adhere well with glass fibers, which increases the strength of the composites. However, higher production costs limit their widespread use [166].

Epoxy resins have superior mechanical and chemical properties. Their ability to cure at both ambient and elevated temperatures makes them a versatile material. Epoxy resins also have a high glass transition temperature and good adhesion to a wide range of materials. On the other hand, epoxy resins have negative properties such as poor crack resistance, brittleness, and low UV resistance. The disadvantage of epoxy resins is the high cost of production, but they are still used in sectors that require the highest quality materials [167].

Although epoxy resins are more expensive than other thermosetting plastics, they have a higher durability with a wide range of applications [167].

To summarize, polyester (i), vinylester (ii), and epoxy (iii) resins exhibit distinct differences in their respective properties (Table 10), applications, production, and cost; (i) is the cheapest and easiest to process, but its recyclability and strength are limited; (ii) offers better chemical resistance and less shrinkage, but it is more expensive; and (iii) is characterized by superior strength and versatility, but its high price and long curing time are major limitations. These differences affect their use in a wide range of industries, from construction and transportation to aerospace and energy.

Moreover, the bonding strength is not solely determined by the type of resin used but also depends on the preparation of the concrete substrate. Al-Rousan et al. conducted a study on the effect of concrete surface preparation on the bond strength of CFRP composites. They showed that the best bond and slip strength were obtained with a brushed surface with holes. In addition, the length of the CFRP sheet has a greater influence on the strength than the width [168]. Similar findings were presented by Szewczak [169], where an increase in adhesion was observed between the sandblasted concrete substrate and the CFRP tape. Furthermore, modification (sonication) and the use of resin additives (microsilica and carbon nanotubes) affected resin viscosity and the potential for greater penetration of the adhesive into the substrate.

The optimum viscosity of the epoxy resin ensures full infiltration without the formation of macropores, while excessive viscosity or too much solvent negatively affects the mechanical properties of carbon nanotube fibers (CNTFs). Additionally, a key step in achieving high composite strength is the defoaming process [170]. In the pultrusion process, resin viscosity has a significant effect on fiber compaction—as viscosity increases, it becomes more difficult to achieve full permeation. Studies have shown [171] that higher viscosity promotes a higher degree of fiber compaction. The optimum viscosity should be within a certain range, but it is preferable to keep it lower, which facilitates soaking at the appropriate injection pressure.

## 5. Research Gaps and Perspectives

In the context of the application of CFRP in composite column structures, the following research questions remain pivotal:

- Can carbon fibers provide an effective alternative to traditional reinforcement in concrete columns?
- To what extent do the perforations provide effective bonding between the concrete core and the surrounding CFRP tube?
- How do the location and size of perforations influence the failure mechanism and its initiation?
- To what extent is it possible that the efficiency of the construction process be improved by using prefabricated CFRP reinforcement elements combined with self-compacting concrete?

The answers to these questions, further explored in the planned research, will provide insights into the potential application of CFRP composites in composite column structures with enhanced load-bearing capacity, such as bridge piers, columns in high-rise buildings, and piles.

To the best of the authors’ knowledge, no publications in the available databases address the use of perforated CFRP composite tubes confined by an outer CFRP tube. To identify existing research, a search was conducted in the Scopus and Web of Science databases using selected keywords (Table 11). In the final stage of the bibliometric analysis, the search scope was narrowed to more specific keywords: reinforcement, CFRP, tube, and perforated, which best aligned with the study’s focus. However, no publications that meet these criteria were identified. Consequently, the search scope was expanded to “all fields”, which yielded a single article by Wang et al. [120], which examines the behavior of perforated GFRP tubes under axial load. Due to its potential relevance to the research topic, this publication has been discussed in detail in Section 3.4.

**Table 11:** Search results for keywords and synonyms [32,172].

Selected Keywords	Number of Phrases	
	Web of Science	Scopus
Step 1		
Reinforcement/strengthening + concrete + CFRP/carbon fiber-reinforced polymer/fiber reinforced polymer/FRP + tube/circular column/hollow section	23	357
Step 2		
Reinforcement/strengthening + concrete + CFRP/carbon fiber-reinforced polymer/fiber reinforced polymer/FRP + tube/circular column/hollow section + perforation/perforated/hole/cutout	0	1
Step 3		
Reinforcement + concrete + CFRP + tube + perforated *	1	0

* Searches expanded to “all fields”.

Therefore, it is justified to explore the use of CFRP not only as external confinement but also as internal strengthening, as this may enhance load-bearing capacity and reduce the risk of FRP softening due to the epoxy resin matrix, which may undergo plasticization at temperatures around 60 °C. Future research will focus on the behavior of perforated CFRP tubes embedded in composite columns. Particular attention will be given to the influence of composite thickness and perforation patterns on the stress–strain response, load-bearing capacity, and damage propagation characteristics of the tested elements. The experimental program will compare two perforation schemes, different slenderness ratios, and the efficiency of internal strengthening—by embedding one or two perforated CFRP tubes inside the composite columns. For a more comprehensive analysis, a numerical study will also be conducted to fully simulate the behavior of composite elements with specified geometry and mechanical properties.

## 6. Summary

The dynamic development of civil engineering has led to the increasingly widespread use of composite structures, which combine the beneficial properties of concrete and steel to enable effective confinement of concrete columns. In engineering practice, fiber-reinforced polymer (FRP) materials are primarily applied for strengthening and repairing corroded reinforced concrete elements. Simultaneously, there is growing interest in the scientific literature in employing FRP materials as external reinforcement and as a potential alternative to conventional reinforcement. This interest stems from their high strength/weight ratio, resistance to corrosion, and the potential for reducing construction time.

FRP-strengthened structural elements are particularly applicable in strategic constructions where high load-bearing capacity, stability, and resistance to dynamic impacts are critical. Moreover, systems such as CFFT or NSM provide specific structural and installation advantages, which should be considered based on the intended use, whether in new builds or retrofit interventions.

Based on the analysis, the following recommendations can support practitioners in optimizing the use of FRP reinforcement:

- Material Selection: CFRP is the most effective in terms of mechanical performance and environmental resistance. For less demanding applications or cost-driven projects, GFRP or BFRP may be considered, acknowledging their limitations in durability and water absorption;
- Design Considerations: Engineers should ensure circumferential fiber orientation to maximize confinement. The number of FRP layers should be optimized, as increasing the number of layers does not necessarily result in proportional improvements in efficiency due to diminishing returns with each additional layer;
- Application Method: In retrofitting scenarios requiring minimal visual impact and greater protection against vandalism or mechanical damage, NSM is preferred over traditional EBR methods. For new constructions requiring both formwork and confinement, CFFT systems offer efficiency in terms of installation and performance.
- Durability and Fire Resistance: While FRP materials are corrosion-resistant, their performance in fire conditions is limited. Designers should include fire-resistant coatings or additives where required. In addition, attention should be paid to surface preparation and resin selection to ensure long-term bond integrity and structural performance;
- Sustainability and Recycling: Although current recycling methods for FRP are limited, engineers should consider hybrid composites or recyclable formulations when sustainability goals are prioritized.

However, a detailed literature review reveals significant gaps in studies concerning the use of FRP as internal reinforcement in concrete columns, particularly in maintaining adequate composite action. To the best of the authors’ knowledge, no existing publications have addressed the impact of perforations in carbon fiber-reinforced polymer (CFRP) materials placed internally within concrete columns, nor have they analyzed the damage propagation mechanisms initiated by such perforations.

## Figures and Tables

**Table 8:** Effect of concrete compressive strength and number of layers on the effectiveness of CFRP reinforcement.

Laminate Type	FRP Layers	Concrete Surface Preparation Procedure	Compressive Strength of Unconfined Concrete (MPa)	Compressive Strength of Strengthened Concrete in Comparison to the Reference (%)	Ultimate Strain/Strain of Unconfined ε_cu_/ε_co_	Reference
CFRP	1	After formwork removal, the smooth concrete surface was thoroughly ground and a primer resin was applied [106]	20.5	+101.5	3.69	[107]
	2			+179.0	5.46	
	3			+207.8	5.46	
	1		49.2	+60.6	2.29	
	2			+70.5	2.06	
	3			+104.5	3.65	
	1		70.6	+21.3	1.94	[108]
	2			+36.1	2.46	
	3			+59.1	3.58	
	1		82.1	+15.8	1.62	
	2			+19.5	7.82	
	3			+49.0	3.04	
	2	CFRP as lost formwork	36.4	+66.5	6.4	[86]
	4		59.0	+40.7	4.9	
	6		102.5	+28.6	4.3	
	1		70.8	+51.5	3.91	[109]
	3			+167.1	7.02	
	5			+149.9	7.13	
	1		111.6	+23.3	2.53	
	3			+55.5	3.09	
	5			+94.6	4.65	
	1	Prior to the application of the primer, the concrete surfaces were dried, cleaned, and leveled	24.5 ^1^	+56.1	2.4	[110]
			32.3 ^2^	+41.2	2.5	
			36.2 ^3^	+18.4	2.5	
			21.3 ^4^	+98.8	2.0	

Aggregate type: ^1^ brick; ^2^ stone; ^3^ recycled brick; ^4^ recycled stone.

**Table 10:** Properties of selected resins.

Properties	Polyester Resin	Vinylester Resin	Epoxy Resin
Flexibility	Low	Medium	High
Production cost	Low	Medium	High
Chemical resistance	High	Very high	The highest
Curing process	Fast, high styrene emission	Similarly to polyester resin	Longer, more demanding
Main application	Transportation, construction materials	Energy and automotive industry	Industry: aerospace, construction and marine

## Data Availability

No new data were created or analyzed in this study.

## Abbreviations

The following abbreviations are used in this manuscript:

AFRP	Aramid Fiber-Reinforced Polymer
BFRP	Basalt Fiber-Reinforced Polymer
CFCR	Carbon Fiber Coated Reinforcement
CFCT	Concrete-Filled Fiber-Reinforced Polymer-Steel Composite Tube
CFDST	Concrete-Filled Double-Skin Steel Tubes
CFFTs	Concrete-Filled Fiber-Reinforced Polymer Tubes
CFRP	Carbon Fiber-Reinforced Polymers
CFST	Concrete-Filled Steel Tube
CFT	Concrete-Filled Tube
CNTF	Carbon Nanotube Fibers
EBR	Externally Bonded Reinforcement
FRP	Fiber-Reinforced Polymer
FTC	Freeze-Thaw Cycles
GFRP	Glass Fiber Reinforced Polymer
GFRP	Glass Fiber-Reinforced Polymer
HM CFRP	High-Modulus Carbon-Fiber-Reinforced Polymer
HSCFFT	High-Strength Concrete-Filled Fiber Reinforced Polymer
JFRP	Jute Fiber Reinforced Polymer
LCM	Liquid Composite Molding
LRS FRP	Large Rupture Strain Fiber-Reinforced Polymer
NSM	Near Surface Mounted
PEN FRP	Polyethylene Naphthalate Fiber-Reinforced Polymer
PET FRP	Polyethylene terephthalate Fiber-Reinforced Polymer
PVC	Polyvinyl Chloride
RTM	Resin Transfer Molding
SCC	Self-Compacting Concrete
SCRIMP	Seemann’s Composite Resin Infusion Molding Process
SMA	Shape Memory Alloy
SRIM	Structural Reaction Injection Molding
UHPC	Ultra-High Performance Concrete
UHSCFFT	Ultra-High-Strength Concrete Filled Fiber Reinforced Polymer

## Nomenclature

$\varepsilon_{cc}$	Peak axial strain of confined concrete [-]
$\varepsilon_{co}$	Peak axial strain of unconfined concrete [-]
$\varepsilon_{fu}$	Ultimate tensile strain of FRP [-]
$\varepsilon_{je}$	Effective tensile strain of FRP jacket at failure [-]
$\varepsilon_{l}$	Lateral strain (hoop strain) of the FRP jacket [-]
$\varepsilon_{h,rup}$	Actual hoop strain of the FRP jacket at rupture [-]
$\nu_{u}$	Ultimate Poisson’s ratio of FRP-confined concrete [-]
$\rho_{f}$	Volumetric ratio of FRP strengthening [-]
$\sigma_{l}$	Lateral confining pressure applied by FRP jacket [MPa]
$c_{2}$	Concrete strength factor [-]
d	Diameter of the confined concrete core [mm]
$E_{frp}$	Young’s modulus of FRP fibers [MPa]
$E_{f\mu }$	Reference Young’s modulus used in empirical model [MPa]
$E_{l}$	Confinement stiffness factor for FRP [MPa]
$E_{seco}$	Secant modulus of elasticity of unconfined concrete at peak stress [MPa]
$f_{1}$	Confinement strength of FRP [MPa]
$f_{cc}^{\prime }$	Ultimate confined concrete strength [MPa]
$f_{co}^{\;⠀}$	Compressive strength of unconfined concrete [MPa]
$f_{l,a}$	Actual peak lateral confining pressure applied by FRP jacket [MPa]
$f_{l}$	Nominal lateral confining stress [MPa]
$f_{lo}$	Threshold confining pressure [MPa]
$k_{1}$	Strength enhancement coefficient [-]
$k_{2}$	Coefficient of strain enhancement [-]
$K_{l}$	Confinement stiffness [MPa]
R	Radius of the confined concrete core [mm]
t	Total thickness of the FRP jacket [mm]
